# Learning automata based energy-efficient AI hardware design for IoT applications

**DOI:** 10.1098/rsta.2019.0593

**Published:** 2020-09-14

**Authors:** Adrian Wheeldon, Rishad Shafik, Tousif Rahman, Jie Lei, Alex Yakovlev, Ole-Christoffer Granmo

**Affiliations:** 1 Microsystems Group, School of Engineering Newcastle University, Newcastle upon Tyne NE1 7RU, UK; 2 CAIR, University of Agder, Postboks 422 4604 Kristiansand, Norway

**Keywords:** Tsetlin machines, neural networks, artificial intelligence hardware design, energy efficiency

## Abstract

Energy efficiency continues to be the core design challenge for artificial intelligence (AI) hardware designers. In this paper, we propose a new AI hardware architecture targeting Internet of Things applications. The architecture is founded on the principle of learning automata, defined using propositional logic. The logic-based underpinning enables low-energy footprints as well as high learning accuracy during training and inference, which are crucial requirements for efficient AI with long operating life. We present the first insights into this new architecture in the form of a custom-designed integrated circuit for pervasive applications. Fundamental to this circuit is systematic encoding of binarized input data fed into maximally parallel logic blocks. The allocation of these blocks is optimized through a design exploration and automation flow using field programmable gate array-based fast prototypes and software simulations. The design flow allows for an expedited hyperparameter search for meeting the conflicting requirements of energy frugality and high accuracy. Extensive validations on the hardware implementation of the new architecture using single- and multi-class machine learning datasets show potential for significantly lower energy than the existing AI hardware architectures. In addition, we demonstrate test accuracy and robustness matching the software implementation, outperforming other state-of-the-art machine learning algorithms.

This article is part of the theme issue ‘Advanced electromagnetic non-destructive evaluation and smart monitoring’.

## 1. Introduction

Advances in sensing devices have enabled a shift towards the fourth industrial revolution [1]. The large volume of data produced by these devices is pushing the technology front of a new generation of artificial intelligence (AI) for Internet of Things (IoT) applications. These applications are expected to make important decisions in the real world instantaneously rather than offloading data to the cloud servers [2]. Such a step change in technology requires significant strides in *energy efficiency*, which continues to be a primary design challenge for IoT hardware designers [3–5].

Existing AI systems predominantly follow the principle of neural networks (NNs). Originally inspired by Rosenblatt's neural automaton in 1957 [6], modern NNs have evolved in complexity across different application domains. Typically, NNs define a learning problem by finding the weighted sum of all inputs in the training phase, organized in multiple layers. The weight updates are defined by a normalized activation function and are performed through rigorous gradient descent exercises. When implemented in hardware, the modular electronic neurons require arithmetic-heavy circuits, such as multiply–accumulate (MAC) units. The number of these units can quickly grow with more inputs and added complexity of the learning problem [7]. Given such a scale of arithmetic complexity, achieving required energy efficiency and performance in NNs can be daunting, which is exacerbated further by the large volume of data generated by IoT devices [8].

Over the last two decades, significant progress has been made in energy-efficient NN hardware research. A vast majority of existing works have considered pruning arithmetic complexity to save energy by exploiting the natural resilience of AI applications to minor deviations or error. Examples include precision scaling [9,10], approximate logic designs [11–13], new analogue or mixed-signal circuit designs [14] and hardware/software co-design for NNs [15]. Recently, there are overwhelming interests in moving away from arithmetic to using binary logic as the core building blocks. Binarized neural networks (BNNs) are an example of this development. The key goal is to condense advanced AI workloads with low-energy footprints. However, this can make the learning process (i.e. accuracy and convergence) sensitive to how gradient descent is designed, which is still arithmetic based [16].

Learning automata, originally defined by Mikhail Tsetlin in the 1960s, constitute another class of machine learning (ML) algorithm that reinforces current action using past history. Each action follows the trajectory of a probability distribution which is updated based on the environmental response the automaton obtains by performing a particular action. As the number of actions and their probability distribution trajectories can have a very large number of combinations, adopting learning automata to ML hardware has been challenging [17,18].

Recently, the Tsetlin machine has been proposed as a promising machine learning (ML) algorithm based on learning automata. The Tsetlin machine simplifies the traditional learning automata by discrete-step action updates through Tsetlin automata, defined as the finite automata with linear tactics. For action updates, each Tsetlin automaton uses rewards for reinforcing an action and penalties for weakening the automaton confidence in performing the action. This discretization with linear step updates allows for formulating the learning problem using powerful propositional logic [19]; furthermore, it simplifies the learning mechanism, enabling efficient on-chip learning. The input data in a Tsetlin machine are encoded in binarized form as a set of propositional logic variables, called literals. These literals are used to build the logic expressions corresponding to inference classes through ensembles of parallel Tsetlin automata, called *clauses*, during training [20]. When training is completed, the inference outputs are described by binarized classifications.

The logic-based structure of Tsetlin machines provides opportunities for energy-efficient AI hardware design. This will require addressing the major challenges of the systematic architecture allocation of low-level resources as well as parametric tuning and data binarization, which cannot be achieved by using high-level synthesis or hardware-assisted acceleration tools. This paper provides the first insights into an AI hardware architecture design using learning automata, addressing the challenges above. Specifically, we make the following *contributions*:

— a new AI hardware architecture capable of on-chip learning, targeting primarily an application-specific integrated circuit (ASIC) implementation;
— a binarization method for encoding data for the proposed architecture;
— an exploration and automation design flow for faster hyperparameter search and hardware optimization using a runtime-reconfigurable field programmable gate array (FPGA) prototype;
— extensive validation experiments using several ML datasets, showing comparative analysis of performance, energy and learning efficacy.

Our aim is to corroborate the principles of learning automata applied in energy-frugal AI hardware design. As such, we will validate the efficiency of the hardware architecture using IoT- scale datasets that are carefully chosen to investigate both single- and multi-class applications as well as to study the impact of noisy inputs on the overall learning efficiency. The remainder of this paper is organized as follows. Section 2 introduces the core learning automaton algorithm, leading to the Tsetlin machine. Section 3 presents the design flow and resulting Tsetlin machine hardware architecture. Section 4 discusses the experimental results using the ASIC implementation, while §5 reports further results from ML experiments conducted on the FPGA platform. Finally, §6 concludes the paper and highlights our future work.

## 2. Machine learning using learning automata

**Figure 1.**
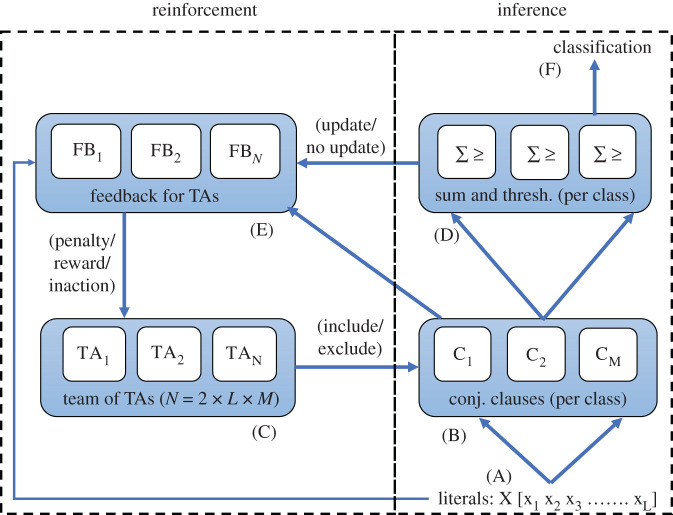
A schematic diagram of discretized implementation of learning automata proposed by Granmo [19]. TA, Tsetlin automaton. (Online version in colour.)

Figure 1 depicts a schematic of different structural blocks in the learning automaton algorithm. The algorithm adopts discrete-step updates using linear tactics, proposed by Granmo [19]. This enables the algorithm to be constrained by a finite number of states, defined by an ensemble of Tsetlin automata. Input data are defined as a set of binarized features and their complements, called literals (A, figure 1). The literals are fed into the learning automaton structure through two major parts: one responsible for inference (i.e. classification) and the other for reinforcement and feedback for learning (i.e. training). In the following, these parts and their parameters are further detailed.

### (a) Inference

**Figure 2.**
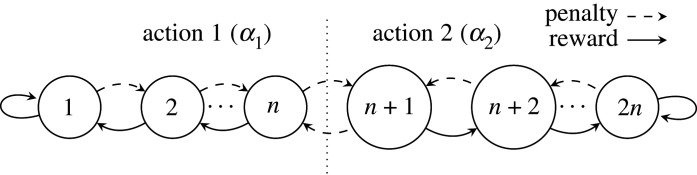
State diagram for the Tsetlin automata.

The main inference component is the conjunctive clause (B, figure 1), which uses propositional logic expressions for output classification. The composition of each clause is controlled by a team of Tsetlin automata, each of which has a pre-defined number of states, divided between actions (figure 2). The automata decide whether their associated literal should be included in the clause or not, following a number of reinforcement steps (§2b).

Each inference class has a set of clauses associated with it. Each clause produces a vote or no vote for its class. Half of the clauses can vote positively, and half of the clauses can vote negatively. Each clause is by itself nonlinear since the clauses are conjunctive. The voting system is linear (summation), followed by thresholding/argmax. The votes are summed to produce a collective result which gives an indication of confidence. This confidence is used to influence future decisions of the automata (E, figure 1).

In a single-class inference problem, the output layer is a simple thresholding function. If the votes are positive (or zero), the input data are determined to belong to the class. For a negative sum, the input data are determined to *not* be in the class. For multi-class problems, we replace thresholding with argmax to determine the output class (D, figure 1). In this case, the class summation becomes indicative of confidence and argmax chooses the class with the highest confidence, thus avoiding any ambiguity in classification.

### (b) Reinforcement

Fundamental to reinforcement are the team of Tsetlin automata (C, figure 1). Such automata are also known as automata with linear tactics to emphasize the fact that they allowed gradual ascent, or reinforcement, in performing a particular action, and equally gradual descent from one action to performing another action. A variety of types of such learning automata have been studied in [21].

In the Tsetlin machine implementation, a two-action Tsetlin automaton is described by the state diagram in figure 2. The automaton may be given a reward, causing it to reinforce the current action decision (e.g. action 1) by moving away from the midstate (i.e. state *n* in figure 2). Conversely it may be given a penalty, which moves the state towards the decision boundary.

In relation to processing the binarized literal through Tsetlin automata within clauses, the two actions are *include* and *exclude*. The update of the automata requires reinforcement through penalty, reward and inaction. The decision on whether to update individual automata is controlled by a number of conditions based on: (i) the values of literals, (ii) votes from clauses described earlier, and (iii) current actions of Tsetlin automata in terms of *include* and *exclude*. For further details of how an algorithm reinforces these updates, refer to [19].

### (c) Parameters

The efficacy of ML using learning automata depends on a number of hyperparameters, which must be carefully tuned before training. The process of tuning for accuracy and convergence for a given problem is called a *hyperparameter search*. Table 1 shows the Tsetlin machine hyperparameters with their associated symbols used throughout this paper. The numbers of binary inputs and classes are fixed by the problem at hand.

**Table 1.** Tsetlin machine parameters and their symbols.

number of binary inputs	*N*_Inputs_
number of classes	*N*_Classes_
number of clauses per class	*N*_Clauses_
number of automaton states	2*n*
automaton decision boundary	*n*
automaton initialization state	*ϕ*_Init_
feedback threshold	*T*
learning sensitivity	*s*

For large ML problems, a software-based hyperparameter search can be computationally expensive, requiring several hours to weeks of iterative computation times. Typically, a software- based hyperparameter search aims to achieve better accuracy and performance. However, hardware objectives are marginally different as hyperparameters need to be carefully exercised in low-level design configurations for energy frugality, while achieving an acceptable accuracy. To achieve these objectives as well as an accelerated search, we will use an FPGA-based hardware prototype on ML problems with different dataset sizes. This prototype will also be used for automating the process of faster design exploration, while managing the trade-offs between power, performance and efficacy.

## 3. Proposed hardware architecture

**Figure 3.**
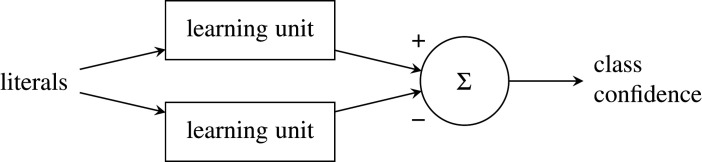
The basic architecture of a two-clause Tsetlin machine classifier.

The hardware architecture inspired by the Tsetlin machine implementation of learning automata (presented in §2) is designed by exploiting the principle of *maximal parallelism*. An update of all Tsetlin automata and computation of all clauses is executed in parallel. This allows for processing one datapoint (which is a set of the input features concurrently updating all automata in the Tsetlin machine) in a single clock cycle. Figure 3 shows the basic Tsetlin machine inference (i.e. classifier) architecture. This block is duplicated for the number of required classes.

**Figure 4.**
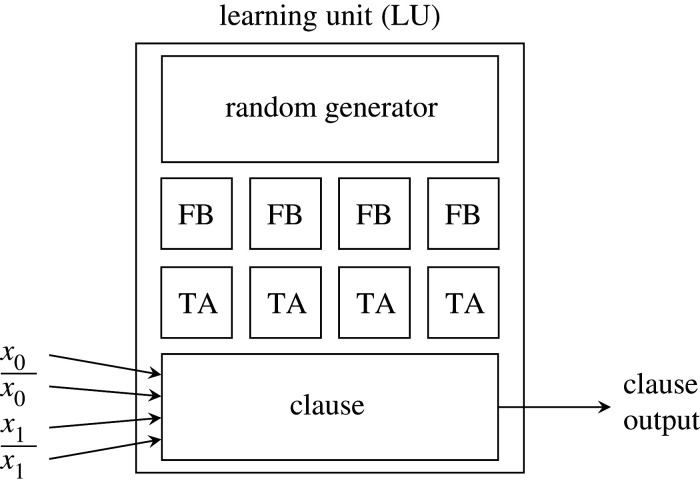
A two-input Tsetlin ML unit comprising a clause, Tsetlin automata (TA), feedback (FB) and a random source.

The input to the proposed architecture is a set of binarized Literals. The Literals are organized in pairs of originally encoded binaries and their inverses (i.e. $\text{Literals}=\{\text{Inputs},\bar{\text{Inputs}}\}$). The learning units (figure 4) are self-contained and include the automata (TA), feedback generation (FB) and random generation associated with one clause. For inference, only the clause computation itself and include states previously calculated by the automata are required; the rest of the learning unit can be omitted or turned off. In the following, different architectural components are described further. For ASIC synthesis, Tsetlin machine parameters are fixed at the compile time, enabling the lowest area and power possible. For FPGA prototyping, hyperparameters can be adjusted on-the-fly for rapid exploration and optimization.

### (a) Binarization

**Figure 5.**
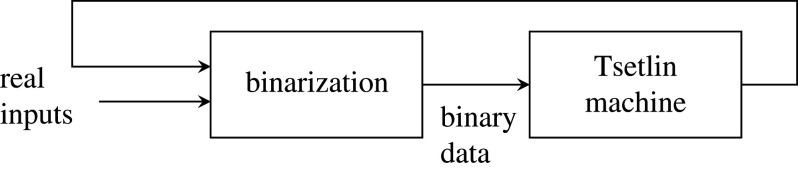
A closed-loop binarization method with accuracy feedback.

The input Literals are encoded in a binarized form before they are compatible in the proposed architecture's learning and inference steps (§2). This binarization process affects the system accuracy; therefore, to tune the accuracy to the required level, binarization is included in the feedback loop during training, as figure 5 shows. Using a suitably chosen encoding method, the raw data are encoded with increased binary precision until the required accuracy is achieved.

The existing encoding method uses pre-defined thresholds and precision levels for encoding the raw dataset [19]. This method ignores the statistical significance of data, which defines how output inference classes are correlated with the dataset. For example, in a multi-class ML problem, it is possible that the majority of the inference classes are statistically orthogonal and independent. A data significance-agnostic method will not exploit this orthogonality towards reducing the binarized encodings, which will lead to over-provisioning of resources (such as the number of parallel automata and the number of clauses) in the hardware architecture.

**Figure 6.**
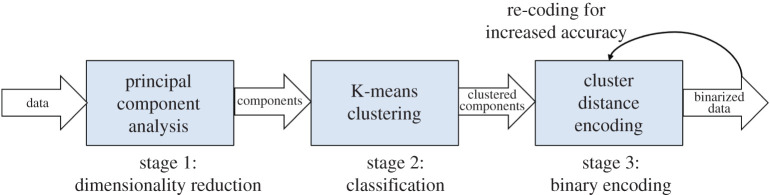
A data flowchart of the significance-driven binarization method, organized in three stages. (Online version in colour.)

For resource-frugality considerations, we developed a significance-driven binarization method consisting of three stages, as shown in figure 6. In the following, we briefly describe these three stages.

*Stage 1* (*dimensionality reduction*). In the first stage, the class labels are stripped off in a given ML dataset and then principal component analysis (PCA) is carried out. PCA generates orthogonal transformation of the dataset to linearly uncorrelated components, defined by eigenvalues that represent their percentage of variance. The outliers in the component definition are discarded, leading to dimensionality reduction.

*Stage 2* (*classification*). After reducing dimensions through PCA, it is possible that the components will still have eigenvalues indicating high correlation between them. At this point, k-means clustering (kMC) is applied to quantitatively differentiate the datapoint positions. We chose kMC as it is a fast and scalable method, which can progressively adapt to cluster centroids starting with a random datapoint [22]. In our approach, we use a hard clustering approach in kMC, which allocates each PCA point to only one cluster. Since we already know the target class each point belongs to from the original class labels, kMC allows for validating their true significance using orthogonality.

*Stage 3* (*binary encoding*). With their true reflection of orthogonality and clusters in stage 2, the distance between the clusters is determined in this stage, leading to our envisioned binarization method. We use a clusters to left, distance from true class, clusters to the right (CDC) encoding scheme that can uniquely define a cluster, maximally maintaining the orthogonality and dimension reductions obtained from PCA. By identifying the cluster distances, threshold points can be estimated from the raw datasets for binary encoding. For overlapped clusters or higher accuracy needs, the thresholds are re-adjusted or re-coded considering the one or more cluster distances, particularly the overlap to the left (C) and that to the right (C).

**Figure 7.**
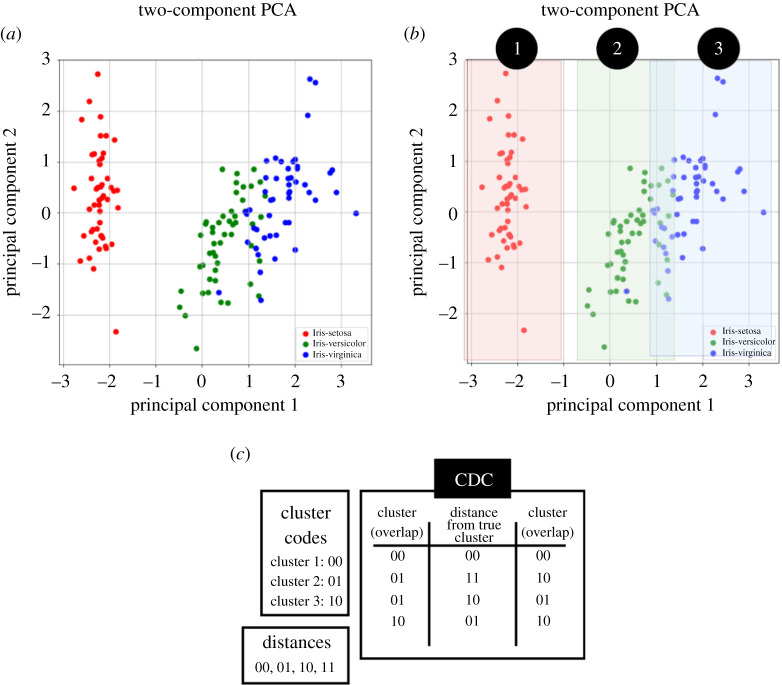
A visual representation of the stage outputs of the data significance-aware binarization method applied to the binary Iris dataset. (*a*) The first two principal components after stage 1. (*b*) The PCA features in two dimensions after stage 2. (*c*) Output binaries generated by the CDC encoding scheme after stage 3. (Online version in colour.)

Figure 7 demonstrates the stage outcomes generated by the proposed binarization method, when applied to the Iris dataset.^1^ Figure 7*a* shows the graphical representation of the first two principal components. We can see that there is a clear classification of the Iris-setosa but an overlap with the other two classes. These classes are then clustered according to their variance, which can be visualized from figure 7*b*. Note how the points seem to be distributed almost parallel to the *y*-axis for each class (particularly for the Iris-setosa class). Figure 7*c* depicts how orthogonal and overlapped classes are encoded in a binarized form for the Iris dataset.

The impact of using a significance-driven binarization method on the machine size cannot be understated. This is because the encoding dictates the number of input features, and the number of automata in the machine scales with 2 · *N*_Inputs_ · *N*_Clauses_ · *N*_Classes_. Following on, the number of automata in the machine is directly proportional to the number of inputs (*N*_Inputs_). Taking the Iris dataset as an example, the existing thresholding technique may produce a 16-bit encoding for data. Our proposed encoding with reduced dimensions and the size of dataset generate a 6-bit encoding with no reduction in inference accuracy, thereby achieving a 2.67× reduction of the machine size.

A full analysis of this method with scalable application to larger ML datasets as well as accuracy-sensitive online optimization of binarization are considered for future work. In this paper, we use pre-binarized datasets as proposed by Granmo [19].

### (b) Reinforcement: Tsetlin automata and feedback

We implement a specialized version of the original Tsetlin automaton described in §2b. We use *α*_2_ = =1 to indicate *include* and *α*_2_ = =0 to indicate *exclude*. We also introduce the notion of *inaction* for the Tsetlin automata. This means it is possible for neither penalty nor reward to be given to the automaton. In our hardware implementation, each Tsetlin automaton is modelled as a counter. The counter stores an internal state in a register which is tuned based on feedback from the current machine state. In the case of inaction, the state remains unchanged. For penalty or reward, the state (*ϕ*) is tuned according to table 2 and saturates according to the bounds 1≤ϕ≤2n,ϕ∈Z.

**Table 2.** Tsetlin automaton internal state tuning.

	include	not include
penalty	−1	+1
reward	+1	−1

The include output is asserted when the automaton internal state exceeds the decision boundary such that include=0 for *ϕ* ≤ *n* and include=1 for *ϕ* > *n*. This indicates that the associated literal will be included in the composition of the associated clause. For our hardware, it is preferable for the number of possible automaton states to be a power of 2 so the include output becomes the most significant part of the state. In other cases, a magnitude comparator would be required at the expense of increased logic area.

Penalty and reward are issued to each automaton based on their associated literal, clause output, include state, summation output, expected class and feedback threshold (see [19] and §2 for further details).

An element of probability is introduced into the state tuning to facilitate diversity of learning among clauses. The probability element is provided by linear feedback shift registers (LFSRs). LFSRs produce a random number sequence in each cycle, which is then compared with a pre-determined sequence to define the probability in the circuit.

After learning is completed in the Tsetlin machine algorithm, the reinforcement logic becomes redundant and only the include state is needed to perform inference. For our ASIC, this means that the Tsetlin automaton registers can be *clock gated* since their contents will not change—that is, the clock signal will be disconnected in order to prevent wasted switching power in the register. Additionally, the feedback and LFSR blocks can be *power gated*, completely removing supply voltage and therefore reducing their quiescent power to zero [23]. These power-saving techniques are essential for enabling maximum inference power and energy efficiency (see §4).

### (c) Inference: clauses, voting and confidence

**Figure 8.**
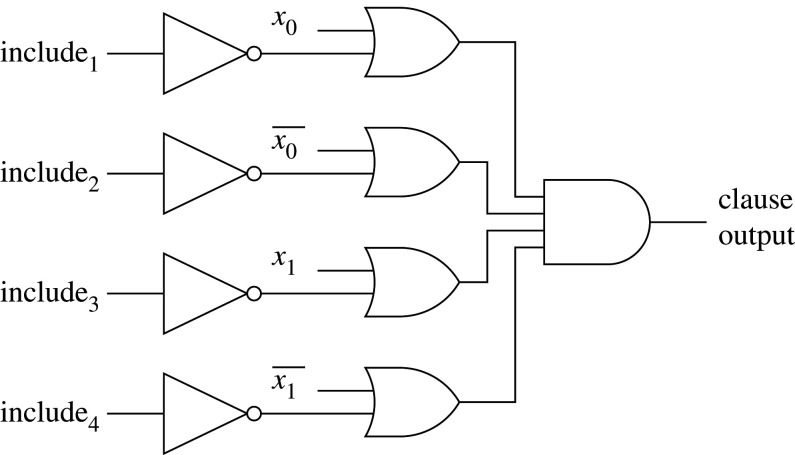
Logic implementation of a two-input clause.

Figure 8 shows the logic for a two-input clause, implemented following the algorithm (§2). The wide and operation is implemented as a balanced tree of and gates for minimum path delay. Include for each literal is used to mask the literal, forcing the corresponding and gate input to 1 if the literal is to be excluded.

**Figure 9.**
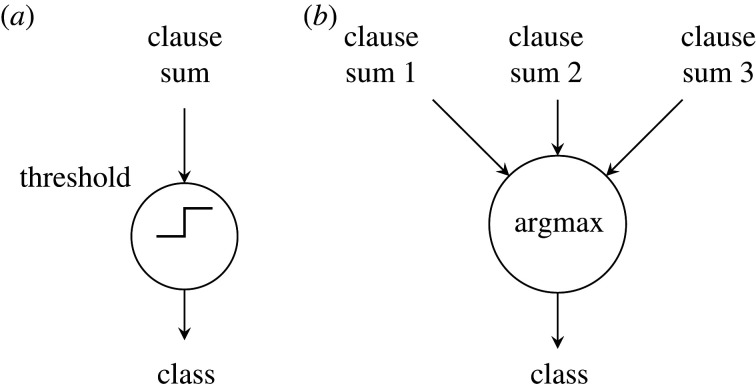
Output functions for (*a*) single-class and (*b*) multi-class Tsetlin machines.

**Figure 10.**
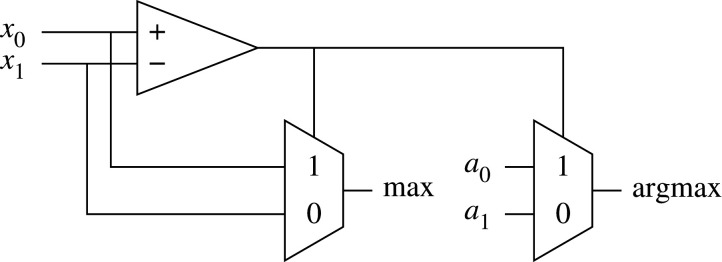
Logic implementation of a two-input argmax.

The simplest Tsetlin machine consists of a single class and uses a threshold function to determine whether the input is in the class or not (figure 9*a*). As discussed in §2a, a multi-class Tsetlin machine can be implemented by instantiating multiple Tsetlin classifiers and choosing the class with the greatest confidence using an argmax block (figure 9*b*). Argmax is built using a tree of comparators with accompanying multiplexers which pass through the corresponding argument number. Figure 10 shows the logic implementation of a two-input argmax which is used to build argmax of higher-numbered input. Max and argmax outputs become ${\text{x}}_{i}$ and ${\text{a}}_{i}$ inputs for the next stage, respectively.

**Figure 11.**
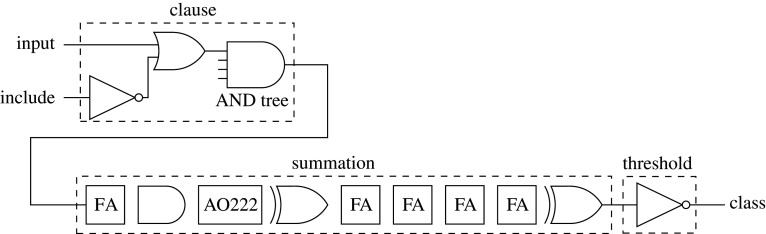
Critical path of the inference hardware.

In figure 11, we investigate the critical path of the inference hardware—using the hardware generated for the noisy xor problem specifically. Include states for the clauses are already calculated by the automata and do not change once learning has ceased. The path is fully combinational and has a propagation delay of less than one clock cycle.

### (d) Design exploration and automation

Typically, a software-based hyperparameter search aims to achieve better accuracy and performance [24]. A hardware hyperparameter search is marginally different as it exercises these parameters in low-level design configurations for energy frugality, while achieving an acceptable accuracy. As such, we developed an FPGA-based automation platform that we can use to flexibly program the hardware to enable accelerated design validations as well as energy frugality and matching stochasticity using small and readily-available datasets. This will ensure that the parameters can be transferred exactly over to the ASIC design and as such will be especially important once we develop hardware-centric Tsetlin machine algorithms which further depart from the software implementation. The FPGA hardware expedites the hyperparameter search process owing to high parallelism of the implementation and is capable of running one training cycle in a time scale of the order of seconds, compared with several minutes for the software implementation on a desktop computer.

**Figure 12.**
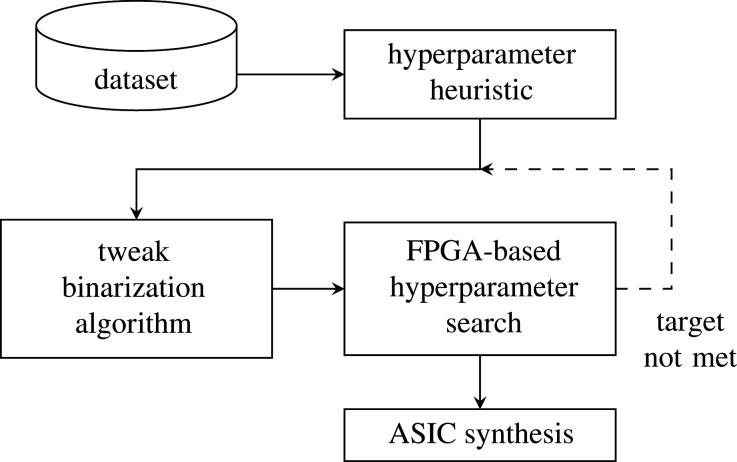
Design exploration and automation flow using an FPGA.

Figure 12 shows the design flow. Initially, there is some heuristic to choose the hyperparameters based on the number of binary inputs. From there, we perform a hyperparameter search which includes a feedback loop to suitably minimize the hardware resources for energy frugality, while also maintaining a high level of accuracy. The final hyperparameters are then used for ASIC synthesis.

An Altera Cyclone V FPGA development board is connected to a host PC through USB connections. A script on the host PC controls the FPGA hardware via Intel Quartus software. The script manipulates the FPGA inputs and outputs (IO) to load the dataset and control the runtime-reconfigurable hyperparameters. After each training cycle the test accuracy is measured and recorded. The hyperparameter search is exhaustive based on a list of possible hyperparameters provided by the user. After the hyperparameter search is complete, the user can choose the preferred hyperparameter configuration based on the accuracy achieved and the resource required for the specific implementation. Changes to the binarization method can be made at this stage if the user's requirements are not met. After the final hyperparameters are chosen, these can be input into the final ASIC synthesis by means of Verilog parameters.

For hyperparameter optimization, we instantiate a parametrizable Tsetlin machine design on an FPGA and use runtime reconfiguration to disable functional units on-the-fly. This methodology allows us to test many Tsetlin machine configurations in a short time and without resynthesizing or reprogramming the FPGA hardware. Clauses can be disabled by forcing their output to zero, meaning they have no effect on Clause Sum. Their associated automata can be disabled to reduce power by giving constant *inaction*. Our future work includes designing the argmax block that can ignore certain inputs to disable the corresponding class, enabling problems with a varying number of class outputs to be optimized on the same FPGA hardware.

**Figure 13.**
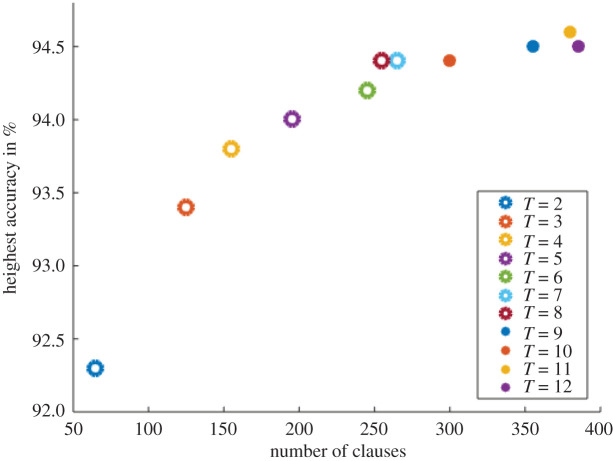
Effect of *N*_Clauses_ and *T* hyperparameters on test accuracy after training for 100 epochs. (Online version in colour.)

Clauses are the main building block of the architecture and also determine the number of automata required in the system. Therefore, our main optimization goal for area and power is to minimize the number of clauses. Overall accuracy of the machine depends heavily on the interaction between *N*_Clauses_ and *T* hyperparameters. The *T* parameter makes almost no difference to the hardware size or power, and we therefore optimize *T* in order to retain as much accuracy as possible with minimum *N*_Clauses_. By using an iterative heuristics algorithm, we have been able to reduce the total number of clauses (given by *N*_Classes_ · *N*_Clauses_) in the Tsetlin machine from 300 to just 60, while retaining a test accuracy greater than 92%. This 80% saving in clauses translates almost directly to the same saving in resources for the ASIC implementation. Figure 13 shows the highest test accuracies achieved for different combination of *N*_Clauses_ and *T*. It is seen that the larger the *T* value, the greater the potential to reach a higher accuracy. However, as *T* increases, so does the number of clauses needed to achieve the highest accuracy.

## 4. Performance and energy efficiency

We test the hardware Tsetlin machine using both 65 nm ASIC technology with a 1 V nominal supply voltage and FPGA synthesis for Altera Stratix IV. For our validation experiments, we use widely available ML datasets (also used in [19]) to train our hardware (more details follow in §5). The noisy xor dataset comprises 12 inputs and one class output. This dataset illustrates the robustness of the Tsetlin ML algorithm and can be used as a first test to ensure functionality with a modest hardware size. Table 3 presents the noisy xor results *post-synthesis* (without performing any layout) for ASIC and FPGA constrained to 100 MHz clock frequency. The binary Iris dataset is a multi-class flower detection task and is representative of IoT applications. For this test, we complete the ASIC layout using Cadence Innovus for a 65 nm low-power technology, giving high-effort power and area figures including scan-chain, IO and clock-tree consumption which we will later compare with other low-power hardware alternatives. We have observed that the energy consumption figures scale linearly with the number of datapoints in the dataset as well as the number of clauses in the Tsetlin machine architecture. More datapoints require extra compute cycles, while additional clauses increase the spatial data processing needs.

**Table 3.** Results of training for ASIC synthesis in 65 nm technology and runtime-reconfigurable FPGA hardware for two different datasets: a single-class noisy xor and a multi-class binary Iris. As expected, the FPGA prototype implementations return significantly higher energy than those of ASIC.

	noisy xor		binary Iris
	ASIC synthesis	FPGA	ASIC layout
*N*_Inputs_, *N*_Clauses_, *N*_Classes_	12, 10, 1		16, 20, 3
ASIC area	0.246 mm^2^	—	0.386 mm^2^
frequency	118 MHz	110 MHz	33.3 MHz
training time	4.24 ms	4.55 ms	1.80 ms
avg. power (training)	16.8 *μ*W	1.10 W	1.85 mW
energy/datapoint (training)	142 fJ	10.0 nJ	55.6 pJ
energy/datapoint (inference)	—	—	30.6 pJ

Noisy xor has a much larger training set (with 5000 datapoints) than binary Iris (with 120 datapoints). However, as only two out of 12 binarized features contribute to the learning formulation it requires fewer training epochs to obtain good accuracy than binary Iris. As the noisy xor implementation features ≈4 × higher clock frequency than binary Iris, both datasets exhibit similar training times. Binary Iris consumes more area, power and energy because of its larger Tsetlin machine structure—requiring *N*_Clauses_ = 20 compared with noisy xor’s *N*_Clauses_ = 10. The FPGA implementation for noisy xor performs similarly to its ASIC counterpart; however, it suffers from high power mainly because of the interconnect overhead.

The logic-based structure in the Tsetlin machine allows for low-complexity, energy-efficient learning and inference. This is a major differentiator when compared with the state-of-the-art NN-based AI. Table 4 compares the Tsetlin machine energy efficiency with three recently reported NN approaches: a mixed-signal neuromorphic approach using time-domain arithmetic organized in a spatially unrolled neuron architecture [25], a low-power FPGA-based convolutional BNN (CBNN) approach that uses exclusive NOR (XNOR) adder-based integer weight biases to reduce the arithmetic-heavy batch normalization for synchronization between the deeper layers [26] and finally an in-memory BNN approach using parallel content-addressable memories (CAMs) to reduce the frequent data movement costs [27].

**Table 4.** Energy efficiency of the proposed Tsetlin machine architecture compared with NN implementations.

	neuromorphic [25]	CBNN [26]	BNN [27]	proposed
technology	65 nm	65 nm	65 nm	65 nm
voltage	1.0 V	1.1 V	1.1 V	1.0 V
features	time domain	no normalization	parallel CAMs	logic based
architecture	spatially unrolled	XNOR based	pipelined	clause selection
infer. energy	48.2 Top J^−1^	25.2 Top J^−1^	88.5 Top J^−1^	**62.7 Top J**^−1^
train. energy	—	—	—	**34.6 Top J**^−1^

Our comparative analysis considered disparities between these approaches in terms of (i) their internal structures in both combinational and sequential parts and (ii) the size of datasets used to validate the efficiencies. To maximally avoid any bias in the presence of these disparities, we normalize the energy efficiency figures in terms of the number of atomic data operations (a set of multiply additions in the case of NNs and and logic followed by argmax in the case of the Tsetlin machine) per unit energy, expressed as Tera operations per Joule, Top J^−1^. The Tsetlin machine energy efficiency is estimated by dividing the post-synthesis energy per datapoint (table 3) by the product (2 · *N*_Inputs_ · *N*_Clauses_ · *N*_Classes_) and then normalizing that to Top J^−1^. As can be seen, the inference Tsetlin machine (which is fully digital) outperforms the highly specialized BNN approaches by up to 2.5 × (62.7 Top J^−1^). This efficiency is enabled by the lean propositional logic within the Tsetlin automaton followed by majority voting between clauses as well as power-gated reinforcement blocks, such as random generation and Tsetlin automaton update circuits. Power gating the key reinforcement circuits causes the slack times to increase significantly, which makes it possible to either improve the inference performance by scaling the operating frequencies up or increase the energy efficiency further by aggressive voltage scaling. The NN approaches depend on parallel binary operations in multiple layers with a set of pre-trained weights and their biases and as such their arithmetic and data movement operations contribute to higher complexities during inference.

The training energy efficiency of the Tsetlin machine is lower (34.6 Top J^−1^) than its inference energy efficiency (table 4). This is because the reinforcement building blocks, such as random generation and Tsetlin automaton update circuits, are now powered on and crucial. It is worth noting here that the Tsetlin machine training energy efficiency is still considerably high (although no training energy efficiency figures were reported for the NN implementations for comparisons). The complexity of Tsetlin machine reinforcement building blocks is significantly lower than the NN approaches, which depend on parallel multiply–add operations in multiple layers followed by gradient-descent-based weight updates. This efficiency during training in a Tsetlin machine can be exploited for emerging IoT applications where continuous on-chip learning is crucial for adapting to environmental changes at the microedge. Our future research includes architectural support for on-chip continuous learning.

Among other comparative examples, Hirtzlin *et al.* [28] achieved 524 fJ per clock cycle in 28 nm technology for the *basic cell* which makes up their BNN architecture including MRAM and registers. This compares with our *clause* building block which achieves 0.661 fJ per clock cycle in 65 nm technology, which however does not contain any memory elements. Another example of work in this area is Benini [8], who claims 0.4 pJ per operation for an NN MAC implemented in 28 nm fully depleted silicon on insulator (FD-SOI) technology. It should be noted that this specialized low-power technology gives a significant advantage over the 65 nm node used in our work. In the area of hyperdimensional computing, Karunaratne *et al.* [29] demonstrate a system in 65 nm silicon capable of 430 nJ *per query*. Here, a *query* is a unit of inference datapoint comprising a natural language sentence. The data structure of each *query* is organized in the form of hypervectors for parallel in-memory interfaces.

**Figure 14.**
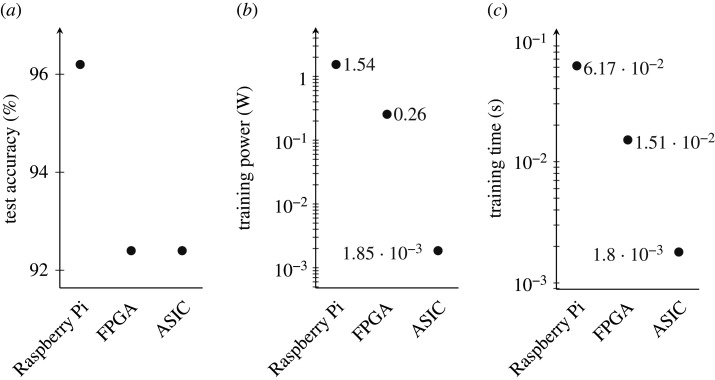
Comparison of software (Raspberry Pi) and hardware (FPGA, ASIC) platforms for (*a*) test accuracy, (*b*) training power and (*c*) training time.

In figure 14, we illustrate the immense power and time advantages of the ASIC Tsetlin machine implementation, compared with more off-the-shelf embedded platforms. We run the same Iris dataset benchmarks across the three platforms: software Tsetlin machine running on a Raspberry Pi 3 (featuring ARM Cortex-A53 cores with 1GB LPDDR2 memory), hardware implemented on a low-power FPGA development board (Digilent Cmod A7-35T) and finally our custom ASIC hardware. For accuracy measurements with a high degree of confidence, we run the training over 100 epochs for all Tsetlin machine implementations. For each experiment, the training times are calculated as the latency per epoch. We measure the power consumption of the software and FPGA-based Tsetlin machine implementations using a precision DC power analyser (Keysight Tech. model: N6705C).

As can be seen from figure 14*a*, the test accuracy of the software is slightly higher than that of the hardware. This is attributed to the differences in random number generation between the platforms. Software random number generation uses significantly higher precision than that used in the hardware implementation, which manifests a more well-defined stochasticity [18]. Both hardware platforms use the same psuedo-random number generation technique (LFSR) and therefore exhibit equal accuracy. According to figure 14*b*, the FPGA platform shows improved power and time over Raspberry Pi since it is free from operating system and extraneous peripheral overheads. The ASIC, however, shows several orders of magnitude lower power consumption again. The ASIC has no reconfiguration overhead or unused logic to leak power. Additionally, logic gates are free to be placed close to each other in order to optimize critical logic path lengths, allowing higher speed computation and faster training/inference times (figure 14*c*). By contrast, the FPGA must configure internal wires to connect the already placed logic gates.

## 5. Machine learning experiments

To observe learning and inference behaviour closely, we ran a number of experiments on the proposed hardware architecture using ML datasets: xor, noisy xor and binary Iris.

**Figure 15.**
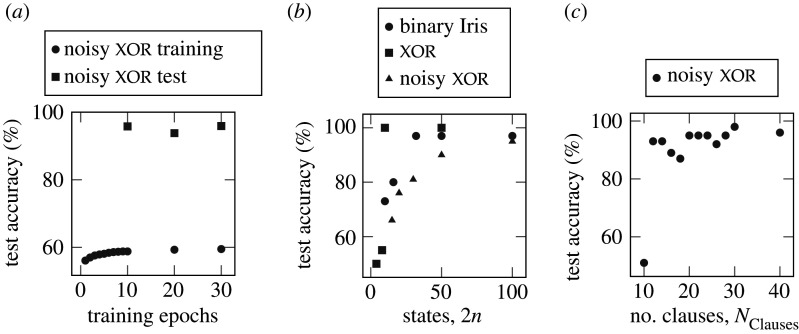
(*a*) Learning convergence of the Tsetlin machine, (*b*) Tsetlin automaton action states versus inference accuracy after 10 epochs, (*c*) Number of clauses per class versus inference accuracy after 10 epochs.

The noisy xor dataset contains 12 binary inputs—two of which are related by xor with the remaining 10 inputs randomized. The training set provides 5000 examples and has 40% of the outputs inverted for added noise; for this reason, training accuracy is limited to 60%. More details of the noise immunity of the Tsetlin machine are available in [30]. The test set provides a further 5000 examples, this time without output inversions, meaning 100% test accuracy is theoretically possible. Figure 15*a* shows the Tsetlin machine achieving 58.8% and 95.8% accuracy in training and test sets, respectively, after only 10 epochs.

In figure 15*b*, we explore how the number of states affects inference accuracy. There is a lower bound on the number of action states (2*n*) for each dataset below which the Tsetlin automata do not provide enough state space to be able to distinguish features of the dataset.

Figure 15*c* shows how the number of clauses per class influences the inference accuracy. For successful learning, the number of clauses must be sufficient to capture the features of the input data and enable an ensemble learning effect. The turning point for learning of the noisy xor dataset is at 12 clauses. As we increase the number of clauses further the accuracy increase tends toward 100% with some variation which is attributed to the stochastic nature of Tsetlin automata feedback.

Table 5 shows how the number of clauses per class influences the inference accuracy. For successful learning, the number of clauses must be sufficient to capture the features of the input data and enable an ensemble learning effect. The turning point for learning of the noisy XOR dataset is at 12 clauses. As we increase the number of clauses further the accuracy increase tends toward 100% with some variation which is attributed to the stochastic nature of Tsetlin automata feedback.

**Table 5.** Comparison of hardware- and software-based Tsetlin machines and XGBoost in the Iris dataset. Here, both hardware and software Tsetlin machines use the same configuration (16, 100, 3).

	accuracy (%)	
Tsetlin machine implementation	train	test
software (desktop PC)	97.3	95.7
software (RPi)		96.2
ASIC (this work)	96.3	97.0
XGBoost	98.3	96.7

## 6. Conclusion

The paper presents the first ever AI hardware design method using the principles of learning automaton. The method leverages the natural ability of an ensemble of Tsetlin automata to learn from a set of training data. The overall framework of a collective of Tsetlin automata lends itself to energy frugality (cf. the principle of least action!) for inferences based on Boolean fabric used for solving classification problems. This was the initial hypothesis for our research and this paper corroborates that through our proposed AI hardware architecture for IoT-scale applications. We also demonstrated the advantages of our hardware design method by comparing power, accuracy and performance figures with software Tsetlin machine implementations on a number of embedded platforms as well as recently reported BNN implementations.

Our hardware implementations in the form of an ASIC benefited from a fast design flow using an FPGA prototype. The design flow facilitated a hyperparameter search to achieve energy efficiency, while also retaining a high-level performance and learning efficacy.We believe that the proposed AI hardware architecture is a crucial step towards packing complex AI systems with on-chip learning capability, particularly suitable for applications that require continuous learning. Future work includes the development of a scalable hardware architecture to enable larger ML problems using advanced architectural allocations and very large scale integration (VLSI) design techniques.

## Footnotes

1 https://archive.ics.uci.edu/ml/datasets/Iris

## Data accessibility

This article has no additional data.
